# Experimental studies on effects of diet on *Lawsonia intracellularis* infections in fattening boars in a natural infection model

**DOI:** 10.1186/s13028-018-0378-4

**Published:** 2018-04-12

**Authors:** Christian Visscher, Anne Kruse, Saara Sander, Christoph Keller, Jasmin Mischok, Robert Tabeling, Hubert Henne, Ricarda Deitmer, Josef Kamphues

**Affiliations:** 1 Institute for Animal Nutrition, University of Veterinary Medicine Hannover, Foundation, Bischofsholer Damm 15, 30173 Hanover, Germany; 2 Boehringer Ingelheim Veterinary Research Center GmbH & Co. KG, Bemeroder Str. 31, 30559 Hanover, Germany; 3 Veterinärgesellschaft im BHZP, Veerßer Str. 65, 29525 Uelzen, Germany; 4 BHZP GmbH, An der Wassermühle 8, 21368 Dahlenburg-Ellringen, Germany; 5 Boehringer Ingelheim Vetmedica GmbH, Binger Str. 173, 55218 Ingelheim am Rhein, Germany

**Keywords:** Boar, Diet, Infection, *Lawsonia intracellularis*, Whey

## Abstract

**Background:**

*Lawsonia intracellularis* is one of the most economically important pathogens in swine production. This study tested the hypothesis that the composition of diets for pigs has an impact on the excretion of *L. intracellularis* in a natural infection model.

**Results:**

Fifty boars (~ 90 kg BW) from a SPF-farm with a strict hygiene and management regime for reducing the spread of an *L. intracellularis* infection up to the beginning of the final fattening period were transported, regrouped and randomly allotted to groups of five animals each at the research facility. After a 1-week acclimatisation period groups were fed one of five diets 4 weeks before slaughter. These were either a finely ground pelleted diet (FP) or a coarsely ground meal diet (CM), both consisting of wheat (40.0%), barley (39.3%), soybean meal (16.0%), soybean oil (2.0%) and minor components. In the other meal diets parts of wheat, barley and soybean meal were substituted either with 22% cracked corn (CORN), 16.9% dried whey (WHEY) or 30% raw potato starch (RPS). The animals had a comparable serological status in a blocking-ELISA immediately before the start and at the end of the feeding experiment. Values increased significantly during the trial. In all subgroups (FP/CM/CORN/WHEY/RPS), shedding was detected in week 0 (genome equivalents = GE; log_10_ GE *L. intracellularis*/g faeces: 2.46 ± 2.64/3.58 ± 2.54/3.43 ± 2.37/2.30 ± 3.16/2.58 ± 2.73). The average number of *L. intracellularis* microbes in faeces during the trial period did not differ between the groups (log_10_ GE *L. intracellularis*/g faeces: 3.40 ± 1.53/3.01 ± 1.41/3.80 ± 1.71/3.98 ± 2.20/4.08 ± 2.13). In animals fed the WHEY-diet, significantly lower counts of *L. intracellularis* were found in the caecal content. The acetate content in the caecum was negatively correlated with the serological results at the end of the trial (r = − 0.36; P = 0.010). Butyrate concentrations in the caecal content were negatively correlated with the number of *L. intracellularis* in the caecum (r = − 0.32; P = 0.023).

**Conclusion:**

Therefore, this study provides preliminary evidence that there might be specific dietary effects on the course of a *L. intracellularis* infection.

## Background

The infection with *Lawsonia intracellularis* is a disease present in all pig-producing countries regardless of husbandry systems [1–7]. In a Europe-wide study, a total of 93% of all surveyed fattening units and 97% of all breeding farms were positive for *L. intracellularis*. For Germany, an even higher prevalence was achieved. About 94% of all fattening farms and 99% of the breeding units investigated had been in contact with the pathogen [8].

The degree of severity of the clinical symptoms is reflected by the level of excretion of *L. intracellularis* in faeces [9]. Increasing pathogen concentrations in faeces are significantly associated with significantly reduced daily weight gains [5]. Excretion of only very few numbers of *L. intracellularis* does not seem to have any effects on daily weight gains [5]. However, the extent of this dependency was lower with increasing dry matter (DM) content in faeces [5]. An increase in one log level concerning numbers of *L. intracellularis* in faeces means the probability of that pig having a lower growth rate by a factor of 1.97 [6]. Especially when more than 10^6^ pathogens per gramme of faeces were detected, this was also a significant risk factor for a lower body weight gain [6].

So far, only two main dietary factors have been investigated more systematically depending on the course of an *L. intracellularis* infection: on the one hand, the effect of packaging (meal versus pellets or crumbles), on the other hand the possible effects of lactic acid-rich diets. On the basis of a survey conducted on 79 Danish pig farms, a reduced in-herd prevalence of *L. intracellularis* was associated with home-mixed and (or) non-pelleted diets [10]. On farms using commercially prepared pelleted diets the prevalence was higher [10]. Therefore, an indirect influence of diet on *L. intracellularis* colonisation would appear possible. This has already been reported in pigs [11, 12] as well as in hamsters [13]. A relative reduction in the numbers of *L. intracellularis* in the total ileal microbiota of pigs fed a non-pelleted diet could be shown [11]. Overall, an effect of diet on the course of an experimental *L. intracellularis* infection was found [12]. Reproducing the effects with a ‘home-mixed diet’ (coarsely ground, non-pelleted diet) following experimental infection with *L. intracellularis* failed [12]. Feeding a fermented liquid diet (rich in lactic acid) to pigs following experimental infection with *L. intracellularis* delayed the excretion of the organism [12]. Offering a standard diet in the same study (based on wheat, barley and soybean meal) supplemented with 2.4% lactic acid led to limited pathological lesions in pigs. Examination was done 28 days after inoculation [12].

The present study was based on the hypothesis that both, the packaging and the potential of a diet to foster the concentrations of specific fermentation products (lactic acid, butyrate) specifically minimise the effects of *L. intracellularis* infections in pigs. A natural infection model was used because it simulates the conditions in practice more than an experimental infection approach. Transport and regrouping of subclinically infected individuals from a high health herd was the starting point for the model. This was intended to simulate the phenomenon observed under natural conditions [14]; namely, the spread of infection after transporting animals from a high health herd to other herds. This is the first study testing five diets differing in terms of the nature of the ingredients in a natural *L. intracellularis* infection model in fattening boars in the final finishing phase.

## Methods

The study consisted of three stages of sampling, which require an explanation regarding classification in accordance with the German Animal Protection Act or the Council Regulation (EC) No 1099/2009 on the protection of animals at the time of slaughter.

First, serological results from routine examinations of organ samples of boars (*L. intracellularis* serostatus of boars) previously carried out on the supplier farm for boars were provided by the corresponding field veterinarian prior to the trial. The sampling was thus not subject to an approval in accordance with the Animal Protection Act.

Second, before starting the experiments, the feeding trial itself was examined routinely by the Animal Welfare Officer of the University of Veterinary Medicine, Hannover, Foundation, Hanover, Germany. This evaluation categorically stated, that the study was not based on an animal experiment requiring notification or approval in accordance with the Animal Protection Act.

Third, at the end of the trial, the animals were anaesthetised using a new method. This method required notification and approval from the relevant authority (exemption: file 32.22.2, Department of Law and Order, state capital of Hanover). The slaughtering process took place at the abattoir in Hannover. The pigs were subsequently killed by bleeding after finishing the trial.

### Natural infection model, animals and housing

Fifty boars from a specific pathogen-free (SPF) pig fattening unit were chosen for the experiments. The animals were selected at a specific date. At ~ 90 kg body weight (BW) at the farm, already 50% of the animals had been serologically positive for the pathogen in the blocking ELISA (PI value ≥ 30) in the past. This was also the case in the experiment. At this time, it can be assumed that an intensive pathogen exchange can be expected within the following few weeks. In particular, if transport and regrouping additionally take place. Under these circumstances, it can be assumed that the pathogen excretion is high, especially if the herd of origin has a high health status and the infection occurs late [14].

The animals were transported to the Institute for Animal Nutrition, University of Veterinary Medicine Hannover.

The 50 boars from the German Federal Breeding Programme were genetically defined animals of five lines with regard to inheritance factors for androstenone and skatole. Groups of five boars each were newly formed, with one representative of each genetic line per group. The boars were kept in groups of five animals each (Table 1) in 2 × 3 m boxes with solid floors. Each box had two feeding troughs, each 1 m in length. Chains and playing balls were offered as environmental enrichment material. Covered metal pits, each 20 cm wide, were placed at the back of the boxes.

**Table 1:** Experimental design concerning number of animals, repetitions and group size

Item	Treatment				
	FP	CM	CORN	WHEY	RPS
Group size	5	5	5	5	5
Repetitions/treatment	2	2	2	2	2
Total no. of animals	10	10	10	10	10

*FP* fine pelleted diet, *CM* coarse meal diet, *CORN* meal diet with 22% cracked corn; *WHEY* meal diet with 16.9% dried whey, *RPS* meal diet with 30% raw potato starch

### Feeding regime, performance parameters and sampling

The experiments were carried out for a period of about 5 weeks, this being divided into a 1-week acclimatisation phase and a 4-week experimental phase. During the 1-week acclimatisation phase (Time point 1 = TP1), the boars were given a pelleted diet (Vereinigte Saatzuchten Ebstorf-Rosche eG, Ebstorf, Germany). This diet had previously been fed on the farm. The five complete diets used during the 4-week trial period (TP2–5) had been produced at the research institute itself. These differed from one another either in their composition or in their packaging (Table 2). In each case, two boxes received one of the five compound feeds used in the experiment. The grinding of the barley and the wheat was carried out in the hammer mill for all groups. For the control group (fine pelleted = FP), a 1 mm sieve insert was used, whereas for the other groups either a 6 mm sieve insert (coarse meal = CM) or a 3 mm sieve insert (corn = CORN, whey = WHEY and raw potato starch = RPS) was selected for preparing the diet. Further processing (pelleting) was done for the control group (FP). All other groups received the diet in meal form ad libitum. The amounts of feed consumed were recorded on a daily basis. Quantification was done at group level (n = five animals/group). The animals always had free access to drinking water.

**Table 2:** Ingredient and nutrient composition of the experimental diets offered in the experimental period

Item	Diet	FP	CM	CORN	WHEY	RPS
Diet composition (%)						
Wheat		40.0	40.0	27.7	32.4	23.5
Barley		39.3	39.3	30.0	32.2	19.0
Soybean meal		16.0	16.0	16.7	15.0	15.0
Corn		–	–	22.0	–	–
Whey powder		–	–	–	16.9	–
Potato starch		–	–	–	–	30.0
Wheat gluten						6.00
Soybean oil		2.00	2.00	1.00	1.50	3.00
l-Lysine		0.30	0.30	0.20	0.20	0.30
Methionine		0.10	0.10	0.10	0.10	0.10
Salt		0.30	0.30	0.30		0.30
Monocalcium phosphate		–	–	–	–	0.80
Mineral feed^a^		2.00	2.00	2.00	1.70	2.00
Sodium chloride		0.30	0.30	0.30	0.30	0.30
Analysed nutrient composition (g/kg DM)						
ME (MJ/kg DM)		15.6	15.5	15.5	15.2	16.1
Crude ash		46.8	46.7	46.5	50.6	44.8
Crude fat		41.7	42.1	34.8	33.1	50.2
Crude fibre		34.5	40.0	36.2	33.6	28.3
Crude protein		192	187	187	181	189
Starch		482	487	526	405	546
Sugar		44.1	39.5	39.1	125	31.0
Ca		6.90	7.15	6.95	7.45	8.65
P		4.40	4.45	5.10	5.55	5.85
Mg		1.90	2.00	2.05	1.90	1.65
K		6.90	6.70	6.95	10.7	5.60
Na		2.60	3.00	2.65	2.50	2.55
Lysine		13.8	13.5	12.7	12.5	12.9

*FP* fine pelleted diet, *CM* coarse meal diet, *CORN* meal diet with 22% cracked corn, *WHEY* meal diet with 16.9% dried whey, *RPS* meal diet with 30% raw potato starch

^a^Supplement containing (per kg): Ca 24.50%; P 2.40%; Na 5.10%; Lysine 6.60%; Methionine 1.50%; Threonine 1.00%; 450,000 IU vitamin A; 50,000 IU vitamin D3; 2950 mg vitamin E; 4000 mg Fe; 3500 mg Mn; 3500 mg Zn; 600 mg Cu; 60 I; 13 mg Se; 5 mg Co; 20,000 IU phytases

Body weight of finishing boars was measured individually on the day of delivery as well as at the start and at the end of the four-week trial period with a pair of mobile scales (WA 200, Meier-Brackenberg GmbH & Co. KG, Exterlal, Germany). Body weight gain and feed intake on group basis were used to calculate the feed conversion ratio (FCR) per kg gain.

Once a week, samples from all individual animals were collected during defaecation or directly from the rectum. One part of the sample was used for analysis (DM), another part being frozen for further analyses (volatile fatty acids, *L. intracellularis* qPCR).

At the end of the 4-week experimental period (TP6), the animals were killed in a mobile chamber by means of a CO_2_:N fumigation (30:70) at the abattoir in Hanover. The slaughtering process was done in small groups. In each group, genetic lineage and feeding variants were equally represented. On the morning before the dissection all the boars were given their respective experimental diets. In order to avoid unnecessary stress for the animals, only those boars were loaded and transported to the abattoir, which were also going to be killed directly afterwards in the chamber.

During the bleeding, blood samples were taken. After the evisceration by the abattoir personnel, the caecum was ligated before at the base of the caecum with a double ligature. Subsequently, the organ was separated with a pair of scissors and removed. The tip of the caecum was opened. The entire contents were collected and cooled until storage for further processing. For the histological examination, a piece tissue from the intestinal wall (about 3 × 2 cm in size) was taken of each boar in the vicinity of the appendix of the caecum. Care was taken to save the taenia-area. The samples obtained from the caecum wall were clamped on cork plates with needles. Afterwards, tissue was transferred to a cup filled with 10% formalin. After a fixation time of about 24 h, the tissue samples were further processed.

### Analytical methods

Diets were analysed by standard procedures in accordance with the official methods of the VDLUFA [15]. The dry matter content (DM) was determined by drying to the weight constancy at 103 °C. The raw ash was analysed by means of incineration in the muffle furnace at 600 °C for 6 h. Determining the crude protein content was done by analysing the total nitrogen content using the catalytic tube combustion method (DUMAS combustion method; Vario Max^®^, Elementar, Hanau, Germany). The crude fat content was determined after acid digestion in the soxhlet apparatus. The content of crude fibre was determined after washing in dilute acids and alkalis. Starch determination was carried out polarimetrically (Polatronic E, Schmidt und Haensch GmbH & Co., Berlin, Germany). The sugar content was analysed using the Luff-Schoorl method by titration with sodium thiosulphate. The mineral content was determined by atomic absorption spectrometry (Unicam Solaar 116, Thermo, Dreieich, Germany). Amino acids were determined by ion-exchange chromatography (AA analyser LC 3000, Biotronic, Maintal, Germany). The content of volatile fatty acids in the homogenised caecal chyme was measured by means of a gas chromatograph (610 Series, Unicam, Kassel, Germany). After the sample had been mixed with an internal standard (10 mL of formic acid 89% and 0.1 mL of 4-methylvaleric acid), the mixture was centrifuged and then subjected to gas chromatography with a column temperature of 155 °C (injector: 175 °C, detector: 180 °C).

The serological tests were carried out using a sandwich blocking ELISA [16]. This ELISA has a specificity of 98.7% and a sensitivity of 96.5% and works with specific monoclonal antibodies. Cut-off values for the blocking ELISA test are given as percent inhibition (PI) with a cut-off value of PI 30.

The mean *L. intracellularis* content was determined from the aliquot of the homogenised faeces via quantitative PCR using established methods [17]. Results are given in genome equivalents (GE) per gramme faeces.

For histopathological analysis, the fixed tissue specimens were processed by routine methods, embedded into paraffin, and sectioned at 2 μm; three series (with three cuts per series) with a minimum distance of 25 μm were prepared and fixed on microscope slides (Superfrost^®^ plus, Menzel GmbH & Co. KG, Brunswick, Germany) per block.

To measure the crypt depth of the caecal epithelium, a hematoxylin–eosin (HE) staining was performed in accordance with a standard protocol (two tissue sections per animal with a minimum separation of 50 μm). For each boar, these two HE stained sections were examined with regard to the crypt depth of the caecal epithelium. The measurement was carried out with a photomicroscope (Axiophot, Zeiss, Oberkochen, Germany) and the program analysisSIS^®^ 3.0 (Soft Imaging Systems, Münster, Germany). For each slide, ten completely cut crypts were measured from their base to the opening to the intestinal lumen.

### Statistical analyses

The statistical analyses were performed using the Statistical Analysis System for Windows SAS^®^, version 9.3, (SAS Institute Inc., Cary, North Carolina, USA). The group comparison for performance parameters (BW, ADWG), results of serological analyses (PI values), quantitative detection of *L. intracellularis* in faeces and caecal content, DM content in faeces, short chain fatty acids in caecal content and crypt depth of caecal wall were performed by a one-way analysis of variance (ANOVA) for independent samples. The Ryan-Einot-Gabriel-Welsch multiple range test was used for the multiple pairwise means comparisons. The distributions of the residuals from the linear models belonging to the analyses of variance were close to the normal one.

In the case of non-normalised data, overall differences in parameters between groups were assessed by the Kruskal–Wallis equality of populations rank test followed by comparisons with a Wilcoxon signed-rank test. The latter was carried out in pairs to investigate differences in the mean values. The transition from the comparative to the experimental-related error probability was achieved by the so-called “α-adjustment” according to Bonferroni.

Differences in concentration between *L. intracellularis* counts at different time points depending on group as well as differences between DM content in faeces between groups were examined by means of one-way analyses of variance for repeated measures.

For the correlation-analysis of data with normal distribution, the correlation coefficient of Pearson was used. In non-normally distributed residuals the Spearman`s rank correlation coefficient was calculated.

All statements of statistical significance were based upon P values smaller than 0.05. This approach controls the comparison-wise error rate.

## Results

The experiment ran as planned. There were no animal losses. Changes in faecal consistency, which were associated with the detection of *L. intracellularis*, were not treated with antibiotics due to the short duration of the clinical manifestation.

The experimental diets could be regarded as almost isoenergetic (15.6 ± 0.33 MJ ME/kg DM) and isonitrogenous (187 ± 4.02 g/kg DM). The lysine contents were comparable (13.1 ± 0.55 g/kg DM) as intended when formulating the diets (Table 2).

### Performance parameters

Overall, there were no significant differences in the final body weight (Ø at group level: 134 ± 3.42 kg), or in the weight development between the groups (Ø at group level: 1227 ± 82.8 g/day; (Table 3). In numerical terms, the performance in the RPS group was the lowest (1104 ± 336 g/day). If, however, independent of the diet, the mean *L. intracellularis* excretion by category during the experimental period showed numerical differences in terms of performance. Animals with an excretion greater than 5 × log_10_ GE/g faeces had a numerically lower ADWG (log_10_ GE/g faeces < 4: 1.246 kg ADWG; log_10_ GE/g faeces ≥ 4 < 5: 1.252 kg ADWG; log_10_ GE/g faeces ≥ 5 < 6: 1.150 kg ADWG; log_10_ GE/g faeces ≥ 6: 1.147 kg ADWG).

**Table 3:** Body weight, average daily weight gain and feed conversion ratio in finishing boars (n = 10/treatment) fed diets with different compositions during the 4-week trial period

Item	Time	Treatment									
		FP		CM		CORN		WHEY		RPS	
		Mean	SD	Mean	SD	Mean	SD	Mean	SD	Mean	SD
Body weight (kg)	TP1 (start)	98.7	6.97	97.0	6.85	99.1	6.77	97.0	7.77	96.1	8.12
	TP6 (final)^a^	138	13.7	135	8.22	136	7.48	133	10.1	129	13.1
ADWG (kg)	Ø TP2–5 (week 1–4)	1.326	0.272	1.273	0.161	1.225	0.164	1.208	0.250	1.104	0.336
ADFI (kg DM)	Ø TP2–5 (week 1–4)	3084		3217		3242		3261		2861	
FCR (kg/kg)	Ø TP2–5 (week 1–4)	2.64		2.87		2.93		2.94		2.94	

*FP* fine pelleted diet, *CM* coarse meal diet, *CORN* meal diet with 22% cracked corn, *WHEY* meal diet with 16.9% dried whey, *RPS* meal diet with 30% raw potato starch, *ADWG* average daily weight gain, *FCR* feed conversion ratio in kg diet per kg weight gain, *Ø* abbreviation for mean value, *TP1* time point 1 (start), TP2 (week 1), TP3 (week 2), TP4 (week 3), TP5 (week 4), TP6 (slaughter)

^a^30 days later; no significant differences between values in a row detectable at P < 0.05

### Blood analysis

The PI values before the start of the experiment (Ø PI values at group level: 29.8 ± 2.77; 50% of animals with PI values ≥ 30) and at the time of slaughter (Ø PI values at group level: 55.5 ± 3.59) did not differ between groups at each time point. There was a significant increase in each single group between PI values from the start of the experiment to slaughter (Table 4). The comparison of delta values in PI values between the start and end-points of the different groups showed no differences.

**Table 4:** Results of serological investigations, analyses in faeces (qPCR *Lawsonia intracellularis* and DM) as well as counts of *L. intracellularis* and concentrations of short chain fatty acids in caecal content and cecal crypt depth in groups of finishing boars (n = 10/treatment) fed diets with different compositions

Item	Time	Treatment									
		FP Mean	SD	CM Mean	SD	CORN Mean	SD	WHEY Mean	SD	RPS Mean	SD
		Blood									
PI values blocking ELISA	TP1 (start)	31.0^B^	12.9	30.5^B^	16.9	25.5^B^	13.1	29.0^B^	14.7	32.9^B^	14.6
	TP6 (final)	53.7^A^	10.3	50.1^A^	10.3	57.9^A^	4.84	57.3^A^	3.92	58.7^A^	7.15
	Δ TP1 to TP6^†^	22.7	18.9	19.6	23.3	32.4	14.2	28.3	16.2	25.8	17.6
		Faeces									
log_10_ GE *L. intracellularis* (per g)	TP1 (start)	2.46^AB^	2.64	3.58^AB^	2.54	3.43	2.37	2.30	3.16	2.58^B^	2.73
	TP2 (week 1)	1.08^B^	2.32	1.54^BC^	2.48	2.52	2.68	3.75	3.51	1.90^B^	2.46
	TP3 (week 2)	4.32^A^	3.16	0.59^C^	1.87	3.06	3.32	4.11	2.99	2.45^B^	3.33
	TP4 (week 3)	3.64^AB^	3.23	5.83^A^	2.36	5.06	3.09	5.05	3.28	7.25^A^	1.44
	TP5 (week 4)	4.55^A^	3.33	4.27^A^	3.35	4.98	3.42	3.11	3.37	5.46^A^	3.85
	Ø TP2–5 (weeks 1–4)	3.40	3.23	3.03	3.25	3.78	3.19	3.95	3.23	4.08	3.59
	∆ (Ø TP2–5−TP1)	0.93	3.14	− 0.57	3.30	0.38	2.65	1.68	3.58	1.51	3.80
DM content (g/kg)	TP1 (start)	237	78.9	247	24.7	254	28.8	248	84.7	240	29.1
	TP2 (week 1)	273^a^	40.9	248^bc^	10.1	266^ab^	24.8	241^c^	19.9	238^c^	32.1
	TP3 (week 2)	259	84.7	238	18.0	257	97.7	245	16.7	214	18.1
	TP4 (week 3)	274^a^	49.2	249^ab^	24.0	246^ab^	17.9	241^ab^	16.8	226^b^	55.4
	TP5 (week 4)	268^a^	27.4	245^ab^	17.7	263^a^	24.6	245^ab^	17.5	222^b^	25.9
	Ø TP2–5 (weeks 1–4)	269^a^	53.0	245^bc^	17.9	258^ab^	51.16	243^bc^	17.2	225^c^	35.4
		Caecal content or rather caecal wall									
log_10_ GE *L. intracellularis* (per g)	TP6	4.34^ab^	3.83	5.46^a^	3.03	5.16^ab^	3.63	1.57^b^	3.32	5.82^a^	3.51
Starch g/kg DM)	TP6	41.4^b^	7.97	164^a^	35.4	136^a^	40.6	85.1^b^	22.4	179^a^	110
Acetate (mmol/kg FM)	TP6	148	6.64	145	8.07	131	14.4	143	6.90	136	27.1
Propionate (mmol/kg FM)	TP6	50.5	3.98	53.8	5.82	51.0	3.03	49.4	2.80	48.6	9.28
Butyrate (mmol/kg FM)	TP6	20.3^b^	3.32	24.8^b^	3.83	21.2^b^	8.38	24.4^b^	6.09	33.7^a^	9.53
Crypt depth caecum (μm)	TP6	482^b^	57.5	473^b^	50.0	499^b^	106	475^b^	66.0	570^a^	189

Upper case letters (A, B) signify differences in columns (vertical) on the level of a specific feeding group between TPs (between TP1 and TP6 for “PI values blocking ELISA”; between TP1–5 for “log_10_ GE *L. intracellularis*”, between TP1–5 for “DM content”) depending on time point at P < 0.05

Lower case letters (a, b, c) signify differences in a row (horizontal) between feeding groups on parameter and TP-level at P < 0.05

*FP* fine pelleted diet, *CM* coarse meal diet, *CORN* meal diet with 22% cracked corn, *WHEY* meal diet with 16.9% dried whey, *RPS* meal diet with 30% raw potato starch, *Ø* abbreviation for mean value, *TP1* time point 1 (start); TP2 (week 1), TP3 (week 2), TP4 (week 3), TP5 (week 4), TP6 (slaughter), *P*I percent inhibition, cut-off values for the blocking ELISA test with a cut-off value of PI 30, *GE* genome equivalents per gramme faeces, ^†^30 days later

### Faecal analysis

The excretion of *L. intracellularis* with the faeces did not differ between the groups at the different time points, nor did the mean excretion in the experimental period. The difference between the mean excretion in the experimental period (TP2–5) and the initial value (TP1) was positive with the exception of group CM. Group CM, however, showed the highest numbers of *L. intracellularis* in faeces at time TP1 in numerical terms. Within some of the groups (FP, CM, RPS), significant differences were seen as a function of time. In the FP group, the most significant excretion was found for TP3 (4.32 ± 3.16 log_10_ GE/g) and TP5 (4.55 ± 3.33 log_10_ GE/g). In group CM, on the other hand, the excretion at TP3 was the lowest (0.59 ± 1.87 log_10_ GE/g), and the highest at TP4 (5.83 ± 2.36 log_10_ GE/g). In group RPS, a significantly higher excretion with faeces was observed at the end of the trial phase (TP4 7.25 ± 1.44 log_10_ GE/g; TP5 5.46 ± 3.85 log_10_ GE/g).

The DM content in the faeces of the boars did not differ at the beginning of the experiment (Ø DM content at group level: 236 ± 16.8 g/kg). At TP2 in groups WHEY and RPS, at TP3 (numerically), TP4 and TP5, the DM content in group RPS was the lowest, whereas at TP2, TP4 and TP5, the DM content in the control group (FP) was highest. When comparing the average DM content during the experimental period (TP2–5), group FP showed the highest DM content in faeces, group RPS the lowest (P < 0.001). Depending on the time, there were no significant differences within each group.

### Caecal content

In the groups CORN and RPS, the *L. intracellularis* genome equivalents were significantly highest (Table 4). In the WHEY group, numbers of *L. intracellularis* in the caecal content were the lowest. The butyrate concentration in the caecal content was the largest in the RPS group, the lowest in the CORN group. In the RPS group the crypt depths were significantly highest.

### Correlations

Very strong negative correlations (r = correlation coefficient) occurred between the PI TP1 and the PI Δ TP1 to TP6 (r = − 0.90, P < 0.001) as well as between the log_10_ GE TP1 and the Δ log_10_ GE TP2–5 − TP1 (r = − 0.85, P < 0.001; Table 5). A strong positive correlation occurred between the log GE at TP5 and the log GE in the caecum content (r = 0.66, P < 0.001).

**Table 5:**
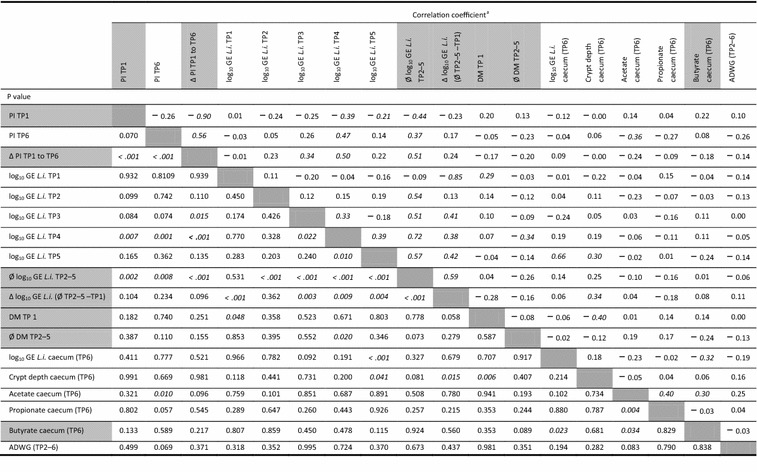
Crosstab regarding intercorrelations between *Lawsonia intracellularis* status, DM content in faeces, volatile fatty acid concentrations in caecal content and ADWG in finishing boars with natural *L. intracellularis* infection

*Ø* abbreviation for mean value, *TP*1 time point 1 (start); TP2 (week 1), TP3 (week 2), TP4 (week 3), TP5 (week 4), TP6 (slaughter), *L.i. L. intracellularis*, *PI* percent inhibition, cut-off values for the blocking ELISA test with a cut-off value of PI 30, *r* Correlation coefficient

*P P* value, statistical significance is based on P values smaller than 0.05 (italics)

^a^Pearson correlation coefficients—for combination of parameters between grey boxes; Spearman’s rank correlation coefficients—combination of unshaded boxes); correlations: 0.00–0.19 “very weak”; 0.20–0.39 “weak”; 0.40–0.59 “moderate”; 0.60–0.79 “strong”; 0.80–1.0 “very strong”

In terms of *L. intracellularis* status in faeces, there were significances between DM at TP1 and crypt depth in the caecum (r = − 0.40, P = 0.006), as well as between the log GE in the caecal content and the butyrate concentration at this location (r = − 0.32, P = 0.023). These correlations were moderate (DM) and weak (butyrate).

At a feeding group level, significant correlations were found between PI TP6 and the mean faecal DM TP2–5 (r = − 0.72, P = 0.020) for group CM as well as positive correlations between butyrate and PI TP6 for the CORN group (r = 0.70, P = 0.024; Table 6). The difference between the PI values correlated significantly negative with the average DM content in faeces of animals in the CM group (r = − 0.74, P = 0.015). The delta PI value correlated positively with the butyrate concentration in the caecal content for the CORN group (r = 0.76, P = 0.010). The counts of *L. intracellularis* (log10 GE) at TP1 correlated negatively with the acetate content in the caecum for the WHEY group (r = − 0.65, P = 0.043). The average log_10_ numbers of *L. intracellularis* during the experimental period correlated positively with the DM at TP1 (r = 0.78, P = 0.012) and negatively with the average DM content (r =− 0.70, P = 0.025) for the FP group during the experimental phase. For the CORN group, the mean log GE content in ZP2–5 correlated positively with the butyrate content (r = 0.68, P = 0.030). For the CORN group, the difference in the log_10_ GE *L. intracellularis* between TP2–5 and TP1 correlated negatively with the average DM content during the experimental period (r = − 0.72, P = 0.018). The *L. intracellularis* counts in the caecal content correlated negatively with the DM at TP1 for the WHEY group (r = − 0.71, P = 0.033), there also being a negative correlation for the RPS group to all volatile fatty acids acetate (r = − 0.74, P = 0.015), propionate (r = − 0.74, P = 0.014) and butyrate (r = − 0.70, P = 0.026).

**Table 6:**
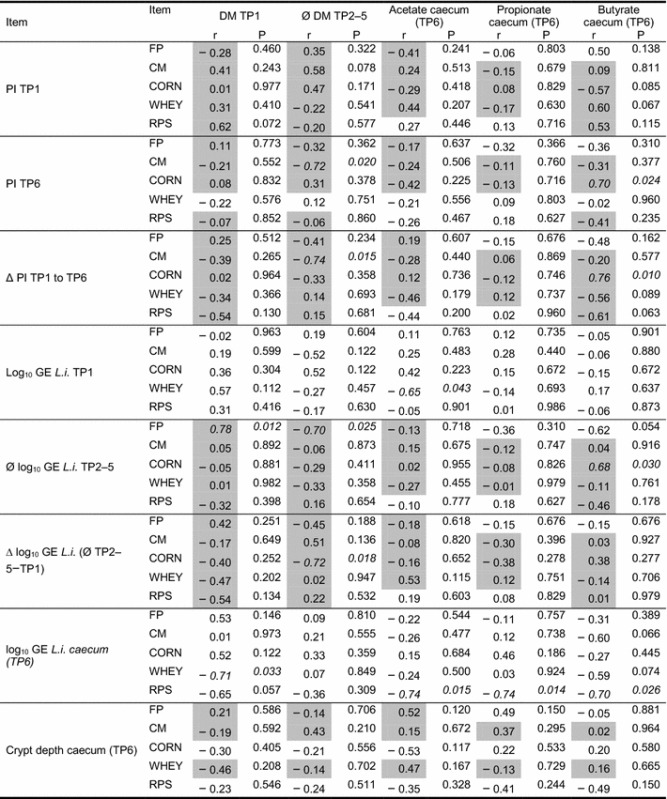
Crosstab regarding intercorrelations between *Lawsonia intracellularis* status, DM content in faeces, volatile fatty acid concentrations in caecal content and ADWG in finishing boars with natural *L. intracellularis* infection

*FP* fine pelleted diet, *CM* coarse meal diet, *CORN* meal diet with 22% cracked corn, *WHEY* meal diet with 16.9% dried whey, *RPS* meal diet with 30% raw potato starch; *TP1* time point 1 (start), TP2 (week 1), TP3 (week 2), TP4 (week 3), TP5 (week 4), TP 6 (slaughter); *L.i. L. intracellularis*, *PI* percent inhibition, cut-off values for the blocking ELISA test with a cut-off value of PI 30, *r* Correlation coefficient

*P* P value, statistical significance is based on P values smaller than 0.05 (italics)

Pearson correlation coefficients—grey boxes; Spearman’s rank correlation coefficients—unshaded boxes); correlations: 0.00–0.19 “very weak”; 0.20–0.39 “weak”; 0.40–0.59 “moderate”; 0.60–0.79 “strong”; 0.80–1.0 “very strong”

## Discussion

In this study, effects of different dietary approaches on the spreading and extent of a natural *L. intracellularis* infection in finishing boars were monitored. The present study is, to our knowledge, the first study on fattening boars, which has analysed the effect of very different diets on the course of a natural *L. intracellularis* infection over several weeks. The experimental conditions were selected in such a way that other factors were minimised. At the same time, different factors were chosen so as to be able to monitor the natural infection. First, boars came from a high health farm with late *L. intracellularis* infections. Second, the chosen animals had a known serological *L. intracellularis* status, 50% of animals being serologically positive in blocking ELISA at the time of transport and regrouping. Third, effects of transport and regrouping were used to provoke the spreading of the pathogen within new groups at the research facility.

### Performance parameters

In the present study, no effect of diet on the performance parameters could be seen. If, however, independent of the diet, the mean *L. intracellularis* excretion by category during the experimental period showed numerical differences in terms of performance. In literature, an effect on the ADWG was seen only for an excretion greater than 10^6^ GE per gramme faeces [7]. However, these previous experiments were conducted with artificial *L. intracellularis* infection. Also, these animals were only 6 or 9 weeks old at the time of artificial infection. In another study on animals of similar age, an effect of the level of *L. intracellularis* excretion on the ADWG (P < 0.001) was shown [5]. The authors of the study emphasise, however, that this effect was particularly important with low DM content in the faeces. In pigs with higher faecal DM (20%) the association between ADG and *L. intracellularis* was minimal. In pigs with faecal DM above 25%, no effect on ADWG was reported [5].

In the experimental period of the present study, the group RPS showed the lowest average DM content in faeces (225 ± 35.4 g/kg). The correlation analysis showed only for the group RPS that the average *L. intracellularis* counts in faeces were significantly negatively correlated with ADWG (r = − 0.78). The present investigations thus confirm that the known relationship applies to RPS. The numerically lowest ADWG in the comparison between the feeding groups was associated with the numerically highest excretion in this group.

In the present study, a negative correlation was observed between the mean values of ADWG of the different feeding groups and excretion levels of *L. intracellularis* between TP2 and TP5 (Spearman`s correlation coefficient: r = − 0.90; P = 0.038). At group average level a higher *L. intracellularis* excretion could be the reason for a lower performance. Nonetheless, also factors such as the palatability of the feed can provoke a different feed intake. Thus, this relation could also be a reason for the numerically different weight development between the different feeding groups.

### Dynamics of infection and diet

The results of the serological tests are clear in the present study. During the acclimatisation and experimental period, in each group an intense *L. intracellularis* spread within the animal group is to be assumed. In each feeding group, a significant increase in the serological response provided evidence of this confrontation with the pathogen. It is known from the literature that the antibodies can fall off already 2 weeks after the excretion peak [9, 18]. This infers that our investigations took place in an intensive infection phase. Antibody titres were high at the end of the trial. This is the best prerequisite for testing a feeding concept in the field.

The haemorrhagic form of an *L. intracellularis* infection is termed proliferative haemorrhagic enteropathy [14]. This form is commonly observed in replacement gilts and boars from high health herds that are introduced onto a new farm site [14]. In the present experiment, the boars had also been obtained from a high health herd. The farmer raised boars of defined genetics from a German breeding company. The animals were transferred to the research facilities and regrouped. In four animals, the acute form of *L. intracellularis* infection occurred during the experiment (divided into groups: 1×FP, 2×WHEY, 1×RPS). These animals showed numerically significant serological characteristics. The initial PI values were lower (20.2 ± 8.74). The final values tended to be higher (59.1 ± 6.69). The ΔPI values were more pronounced during the experiment (∆PI: 38.9 ± 7.39). The average excretion was higher in the experimental phase (TP2–5: 5.86 ± 3.45 log_10_ GE/g) and the ADWG was lower (0.946 ± 0.477). This also indicates the intensive infection in the groups.

Overall, however, during the experimental phase in the present study there was no difference in the quantitative excretion of *L. intracellularis* in faeces as a function of the diet. Feeding of whey powder showed, however, significantly lower *L. intracellularis* levels in the caecum.

In literature, it could be proven that a fermented liquid diet (uncontrolled fermentation for 3–4 days at 24 °C) delayed the excretion of *L. intracellularis* [12]. Furthermore, pigs fed the standard diet supplemented with lactic acid (2.4%) had limited pathological lesions when the intestines were examined 4 weeks after inoculation [12]. High lactate concentrations are also to be expected for the fermented diet. Lactic acid concentration of 5.45% at DM level after an 8 h fermentation process were seen [19]. For a ration with 25% whey on DM basis, an 18–27% share of lactic acid on total organic acids in the caecum was shown [20].

Against this background, for the WHEY group higher concentrations of lactate in the gastrointestinal tract can be presumed. Therefore, it can be assumed that the lactic acid has a certain influence on the level of *L. intracellularis* in the intestine. This could be very promising for future dietetic approaches in swine.

### Correlations

Regardless of the diet`s nature, two significant correlations were identified. First, the acetate content in the caecum was negatively correlated with the serological results at the end of the trial. Second, the butyrate contents in the caecum were negatively correlated with the number of *L. intracellularis* in the caecum. This is a first link between parameters describing the extent of an infection and the substrate properties at the site of the main infection (caecum). In both cases, however, the correlation was weak.

A direct effect is not yet known [21]. However, it is known that the concentration and production of volatile fatty acids in the large intestine is a function of substrate availability for fermentation [22]. Therefore, it depends on feed intake. This, in turn, is lower when animals develop *L. intracellularis* infections. In a US study, a reduction in feed intake (1.27 and 7.85, respectively) in animals aged 28 to 49 days could be observed for different inoculation doses (counts per pig: comparison 1: 7.2 × 10^7^ to 3.8 × 10^5^; comparison 2: 7.2 × 10^7^ to 2.2 × 10^6^) [23]. In pigs at 38–58 days of age a reduction in feed intake of about 3.79% after experimental infection (1.26 × 10^10^ *L. intracellularis* organisms in the inoculum) was demonstrated. As a control animals were treated with 50 ppm tylvalosin for 14 further days after experimental infection [24].

In the present trial, for group FP a positive correlation was found between the DM content in faeces in the acclimatisation phase and the average *L. intracellularis* excretion in the experimental phase. At the same time, however, there was a negative correlation between DM content and *L. intracellularis* excretion during the experimental phase. Animals show more severe clinical symptoms during initial *L. intracellularis* infection [18]. The DM content in the faeces is lower with high excretion of the pathogen [5]. Conversely, this means for our study: The higher dry matter content in the faeces in the acclimatisation phase is indicative of a lower excretion. Therefore, when infected in the experimental phase, these animals will possibly react with a higher level of *L. intracellularis* excretion. During the experimental phase, a negative correlation between the DM content in faeces and the amount of excretion is to be expected. A previous study described that higher excretion is associated with softer faeces [5, 7]. In the present study there was a significant correlation between the ΔPI concerning serological tests and the average excretion in the experimental phase (r = 0.68). The level of excretion is thus also reflected in the extent of the serological response.

For the CM group, the serological response to the infection with *L. intracellularis* was reflected relatively clearly in the faecal consistency. Both, the final PI value and the change in the PI value were negatively correlated with the DM content in the faeces during the experimental phase. An effect of *L. intracellularis* is only relevant to performance when it is also reflected in the DM content [5]. This relationship cannot be confirmed on the basis of the available results for group CM. There was no correlation with the ADWG. Under the conditions of CM feeding the excretion level was too low to lead to performance losses, as described for higher excretion [7].

For the CORN group, there were positive correlations of serological results (PI TP6 and Δ PI) with the butyrate concentration in the caecal content. However, this group was characterised by the numerically lowest butyrate concentrations. At low butyrate concentrations, no positive effect on the excretion of *L. intracellularis* could be observed. Also, in this group there are indications that a clearer response to the pathogen (ΔPI ↑) leads to changes in fecal consistency (softer).

When feeding whey (WHEY), few correlations were detectable. On the one hand, the number of *L. intracellularis* in faeces at TP1 was negatively associated with the acetate content in the caecum. On the other hand, there was a negative correlation between the DM content in faeces in the acclimatisation phase and *L. intracellularis* counts in the caecum. The period between acclimatisation and slaughter is relatively long (more than 30 days). A possible effect can certainly only be achieved if the corresponding feeding regime creates constant conditions in the animal. The measured parameters should also be causally related to the living conditions and characteristics of the pathogen. This is not proven for acetate. For the WHEY group, no correlation between the excretion and DM content in faeces could be determined for TP1 (Spearman: r = 0.57; P = 0.112). It is also known from the literature that high amounts of pathogens in the caecum or adjacent areas need not be found in the colon [25] and therefore could be easily be found in faeces.

The RPS group had the highest concentrations of butyrate and the highest *L. intracellularis* counts in the caecum as well as the deepest crypt in the caecum. For this group a negative correlation of all volatile fatty acids with the *L. intracellularis* content in the caecum could be shown. The PI at TP6 as well as the average *L. intracellularis* content in the faeces were negatively correlated with the ADWG (r = − 0.66 and r = − 0.78), respectively. Thus, the question is whether the disease leads to less substrate or if less substrate favours the disease. The fact that less *L. intracellularis* was detected at absolutely lower butyrate concentrations in the caecal content of the CORN group rather suggests that the butyrate content within this group is more likely to be an indicator function than it being causally related to *L. intracellularis* counts.

## Conclusions

The present investigations indicate that the model of a natural infection in the finishing boars fundamentally works. Therefore, it is a good tool to analyse possible feeding influences on *L. intracellularis* infections. Dietetics can be a starting point for limiting the extent of infection at an early stage. Nevertheless, further tests over longer periods have to be performed first. In this context, feeding concepts with higher original lactic acid contents or provoking lactic acid production, such as from controlled fermentation, could prove promising to limit the effects of an *L. intracellularis* infection on performance and health.

## Abbreviations

ADFI: average daily feed intake
ADWG: average daily weight gains
BW: body weight
CM: coarse meal diet
CORN: meal diet with 22% cracked corn
DM: dry matter
FCR: feed conversion ration
FP: fine pelleted diet
GE: genome equivalents *Lawsonia intracellularis*
*L. intracellularis*: *Lawsonia intracellularis*
ME: metabolisable energy
PI: percent inhibition
RPS: meal diet with 30% raw potato starch
SPF: specific pathogen free. The herd was considered free from infection with Porcine reproductive and respiratory syndrome virus and *Actinobacillus pleuropneumoniae* serotype 1/9/11, 5a/b, 2 (serological confirmed); *Pasteurella multocida* (*free from*—*clinical confirmed*)
TP: time point
VDLUFA: Verband Deutscher Landwirtschaftlicher Untersuchungs- und Forschungsanstalten e. V
WHEY: meal diet with 16.9% dried whey
